# The relationship between music training and cognitive flexibility: an ERP study

**DOI:** 10.3389/fpsyg.2023.1276752

**Published:** 2023-12-07

**Authors:** Jiayi Hao, Yuhuan Zhong, Yazhi Pang, Yuanluo Jing, Yong Liu, Hong Li, Jianbo Li, Maoping Zheng

**Affiliations:** ^1^Key Laboratory of Cognition and Personality (Ministry of Education), Southwest University, Chongqing, China; ^2^College of Cultural Tourism, Chongqing Vocational College of Culture and Arts, Chongqing, China; ^3^School of Psychology, Southwest University, Chongqing, China; ^4^College of Ekistics, Chongqing Vocational and Technical University of Mechatronics, Chongqing, China; ^5^Chongqing Foreign Language School, Chongqing, China; ^6^Chongqing Municipal Educational Examinations Authority, Chongqing, China; ^7^School of Music, Southwest University, Chongqing, China

**Keywords:** music training, cognitive flexibility, oddball, task switching, ERP, N2, P3, N450

## Abstract

**Background:**

Music training involves several cognitive functions in the brain, particularly executive function. Numerous studies have proven a link between the two. Cognitive flexibility is an important component of executive function, however, there has been little study investigating the association between music training and cognitive flexibility.

**Method:**

Music training (*N* = 22) and the control groups (*N* = 26) were included in the present study. A tone-related oddball task was used to investigate the tone-related inhibition and the switch task was to investigate cognitive flexibility. During the switch task, EEG data were collected.

**Results:**

The behavioral results of the oddball task showed that the individuals in the music training group had a shorter reaction time and higher accuracy in both the between-tone and within-tone categories compared to the controls. The behavioral results of the switch task showed similar results, with the music training group exhibiting better reaction time and accuracy performance than the controls. ERP results showed that the music training group had smaller P3 amplitudes and greater N2 and N450 amplitudes than the control group.

**Discussion:**

The findings further supported the link between music training and enhanced cognitive function.

## 1 Introduction

The mastering of an instrument or vocal music requires the integration of multiple sensory and motor processes. Researchers believe that the multi-tasking that music training entails demands facilitation across an arrange of executive functions, which is a set of cognitive processes that involve the ability to plan, organize, and regulate behavior in order to achieve goals. It includes skills such as cognitive flexibility, attention control, working memory, and inhibitory control (Diamond, 2013). George and Coch (2011) found that during an oddball paradigm, musicians exhibited a faster updating of working memory, as evidenced by shorter P300 latency, in both the auditory and visual domains. Additionally, musicians directed a greater allocation of neural resources toward auditory stimuli, as indicated by larger amplitudes of P300 responses. This finding suggests an augmented sensitivity to distinctions between auditory standards and deviants and a reduced cognitive effort associated with updating auditory working memory. The researchers believed that during music training, individuals need to cooperate with their hands, eyes, and brain simultaneously, thus receiving rich training in attention allocation and cognitive regulation. Therefore, the regular practice of playing an instrument or performing in an ensemble may strengthen executive functions, and individuals with such experience would have enhanced executive functions than individuals without (Kiarostami et al., 2022).

Cognitive flexibility is a key concept within executive function, which is defined as the ability to transform in different settings or psychological preconceptions (Chen et al., 2017), and it helps individuals adjust their thoughts and goals more flexibly in their daily and professional lives according to changing needs (Gade and Schlemmer, 2021). Musicians have stronger task-switching abilities and consume less global switch costs than non-musicians, suggesting that musicians have enhanced global processing capabilities and can switch between switching and non-switching components more efficiently (Moradzadeh et al., 2015). In addition, a study found that individuals who received eight sessions of 60-min music training two times per week online could enhance their cognitive flexibility compared to the control group.

The oddball task is a discriminative task triggered by two random stimuli and is used to explore executive functions, including response inhibition and cognitive flexibility (Fichtenholtz et al., 2004). In the task, the oddball stimulus appears infrequently. Participants need to respond to stimuli, distinguish their appearance, and allocate more attentional resources to the rare stimuli (Polich and Margala, 1997). When participants are engaging in the task, they develop a fixed preference for responding to frequent stimuli, which needs to be overcome and transformed into infrequent stimuli (Warren et al., 2011). The switching between tasks is a key evaluative indicator of cognitive flexibility.

Tone-category oddball is an extension of the traditional oddball paradigm, in which participants listen to tones and indicate when they hear a target tone (oddball). Approximately one-third of the world's population uses tonal languages (Yip, 2002). In tonal languages, pitch can convey many subtle emotions and influence the perceptions of individuals, and the response to pitch also represents the development of individuals' executive function. Research has demonstrated that some individuals with poorer tone perception can improve their ability in music perception through music training (Tang et al., 2016). Similarly, even musicians who are not familiar with the tonal language show greater advantages in Mandarin tone recognition tasks and are more sensitive to tone recognition (Lee and Hung, 2008). Therefore, some researchers use the tone-category oddball paradigm to examine the effects of music training on tonal perception ability by comparing task results. Tang et al. (2016) conducted a study on musicians and non-musicians using the tone oddball task and found that musicians are stronger in tone processing than non-musicians. Patel's (2014) research suggested that music training can improve tone recognition skills. Individuals who are trained in music can distinguish language tones with small differences, as well as semitones, which possess even smaller tonal differences. Therefore, the present study examined the executive functions between musicians and non-musicians using the tone-related oddball task.

Previously, many studies have combined ERP data with cognitive flexibility (Kopp et al., 2020). The switch task is a classic paradigm of ERP research and is often discussed with P3, N2, and N450. The memory update theory of P3 suggests that P3 is related to psychological processing, and unexpected or important rare stimuli can cause a spik in P3 amplitudes (Verleger, 1988). When stimuli appear relatively frequently, the amplitudes of P3 remain smaller and consistent. This is due to the fact that more frequent stimuli form longer and lasting memories, while rarer stimuli are accompanied by memory updating and involve more cognitive transformations (Duncan-Johnson and Donchin, 1977; Johnson and Donchin, 1982). Therefore, an increase in P3 may represent achieving task switching (Gajewski et al., 2010). N2 caused by switch task reflects the probability regulation of stimulus categories, with a lower probability causing a larger amplitude of N2 and a higher probability causing a smaller amplitude of N2 (Gajewski et al., 2008; Warren et al., 2011). There are some studies reflecting the relationship between switch task and N450. N450 is a negative wave that reflects an individual's ability to monitor and suppress conflicting information (Szucs and Soltész, 2012). In conflicting situations, N450 will perform more prominently. Overall, the formation and variation of P3, N2, and N450 amplitudes are related to the probability of the stimuli, and switch tasks can cause changes in P3, N2, and N450. A study by Moradzadeh et al. (2015) found that musicians have stronger task-switching abilities and consume less global switch costs than non-musicians, suggesting that musicians have more efficient global processing capabilities and can switch between switching and non-switching components than non-musicians. Research by Kiarostami et al. (2022) also showed that individuals who have undergone music training performed better in task-switching tasks and have better cognitive flexibility than individuals who have not undergone music training.

Previous studies have shown that individuals with music training have advantages in various cognitive abilities, including inhibition response and working memory, but the correlation between music training and cognitive flexibility has yet to be discussed. Numerous studies have shown that the better performance of oddball and switch tasks reflects better task-switching ability and higher cognitive flexibility, which in turn exhibits higher P3 and N2 amplitudes during the tasks (Kopp et al., 2020). Based on the previous studies, we hope to extend the existing findings and reveal the potential relationship between music training and cognitive flexibility through both behavioral and electrophysiological indicators. The study hypothesized that music training is beneficial for the development of individuals' cognitive flexibility. Individuals who have undergone music training will exhibit greater advantages in the switch and oddball tasks, which are reflected in the task performance and the electrophysiological indicators during the tasks.

## 2 Methods

### 2.1 Participants

A total of 48 participants were included in the present study. We categorized 22 participants who majored in music as the music training group. The other 26 participants who majored in other studies and had not received any formal music training prior to the study were categorized as the control group. Participants in the music training group were skilled at one instrument or vocal music; the mean length of music training is 6.14 years (*SD* = 1.58 years), with 1–3 h of practice per day. Each participant reported having normal or corrected-to-normal vision, being right-handed, and having no prior history of neurological or psychiatric disorders. Prior to their participation in the study, written consent was obtained. The study was approved by the Ethics Committee of Southwest University (No. H23089).

### 2.2 The Raven Advanced Progressive Matrices

The Raven Advanced Progressive Matrices (RAPM) is a commonly employed assessment tool designed to measure higher-level general cognitive aptitude and estimate overall intelligence (Bilker et al., 2012). The test comprises a set of 12 pattern-matching questions, each progressively more difficult than the previous. Within each question, participants are presented with a diagrammatic puzzle that lacks a single piece, and their task is to identify the correct missing piece from a provided list of options. Scoring of this test is based on the sum of correctly solved problems, with higher scores indicative of better performance. Notably, the RAPM demonstrates a strong internal consistency, with a Cronbach alpha coefficient ranging from 0.79 to 0.80 (Langener et al., 2022).

### 2.3 Oddball task

**Figure 1:**
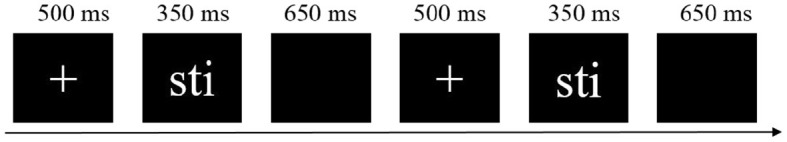
The oddball task used in the present study.

The oddball task (Figure 1) in the present study aimed to explore tone-related response inhibition. The stimuli used were two Chinese syllables (/fa2/and/fa4/) specially constructed for the lexical tone condition. These original stimuli were produced by a female native Mandarin speaker and recorded at a sampling rate of 44.1 kHz. Subsequently, the two speech stimuli were processed in Praat. Apart from the pitch contour difference, other acoustic parameters such as duration, strength, and envelope remained the same. The duration of stimulation was set at 250 ms, including 10 ms rise and fall times, and the sound intensity was set at 70 dB. The above two stimuli were considered as the endpoints of the tone continuum and were used to generate 11 stimuli using the PSOLA synthesis technology as the test material for tone category perception. Prior to the formal experiment, 15 native Mandarin speakers evaluated these 11 sound stimuli. They were asked to judge if each randomly presented stimulus contained two or four tones and responded accordingly by pressing a key. Each stimulus was presented at random for six times, with a time interval of 1,000 ms. Based on the results of behavioral experiments, T3 and T7 were identified as the inflection points of tone category perception. The T3 and the tone probability before T3 were recognized as the rising tone, and the T7 and the tone probability after T7 were recognized as the falling tone. Therefore, T7 was considered as the standard stimulus, T3 as the between-category deviation stimulus, and T11 as the within-category deviation stimulus. Hence, T3 and T7 were used as between-category stimulus pairs, and T7 and T11 were adopted as within-category stimulus pairs for the experimental materials.

The oddball task consists of two blocks. One block includes standard stimuli and between-category stimuli, and the other block includes standard stimuli and within-category stimuli. Each block comprised 500 trials that appeared in a pseudo-randomized order, ensuring that there were at least two successive standard stimuli between deviants. The probability of standards was 90%, while the probability of deviants was 10%. At the beginning of each block, the first 15 stimuli were standard stimuli. Two blocks were presented randomly. The duration of each block was 15 min, with a 5-min rest period between the two blocks. During each trial, a fixation cross “+” would initially appear at the center of the screen for 500 ms, followed by the presentation of 400 ms of speech stimulation and then a blank screen lasting 650 ms, completing a single trial. Participants were required to wear headphones and watch silent movies. They were instructed to press the “F” key when hearing the standard stimulus and press the “J” key when hearing the deviant stimulus. The distance between the movie video and the subjects is 70 cm. The stimulus was presented through binaural headphones with a volume of 70 dB. Subsequently, the participants began to complete the oddball task.

### 2.4 Switch task

**Figure 2:**
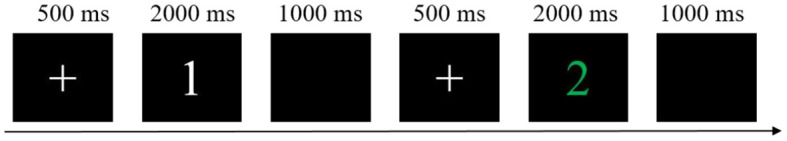
The switch task used in the present study.

The switch task (Figure 2) was designed to explore cognitive flexibility. The stimuli were digits 1–9 (excluding 5) displayed in either white or green color. When the digits were presented in white, participants were asked to compare them with digit 5. They were required to press “Z” when the presented digit was less than 5 and press “M” when it was greater than 5. On the other hand, when the digits were presented in green, participants were asked to judge whether the digit was odd or even. They were required to press “Z” when an odd digit was presented and press “M” when an even digit was presented. During each trial, a fixation cross would appear on the screen for 500 ms, followed by the display of stimuli until the participants responded to the task. If the participants did not respond within 2,000 ms, the stimuli would disappear. After this, a black screen would appear 1,000 ms after the stimulus screen. In the switch task, a repeated trial meant that the task in the current trial was the same as that of the last trial. A switch trial meant that the task in the current trial was different from that in the last trial. The task consisted of two blocks, with each block consisting of 128 trials. In total, there were 256 trials, 128 trials for repetition and 128 trials for switching).

### 2.5 Procedure

All participants completed the oddball task before completing the switch task. Therefore, behavioral results of the oddball task and behavioral and EEG results of the switch task were reported in the present study.

### 2.6 Behavioral analysis

#### 2.6.1 The Raven Advanced Progressive Matrices

An independent sample t-test was carried out to assess the mean RAPM scores of the music training and control groups.

#### 2.6.2 Oddball task

Repeated-measure ANOVAs were conducted on the ACC and the RT, with two groups (music training and control groups) as between-subject variables and two conditions (within-tone and between-tone categories) as within-subject variables. RT or ACC above or below 3 SDs were excluded from the final analysis. In total, 47 participants were included in the final analysis for the oddball task.

#### 2.6.3 Switch task

Repeated-measure ANOVAs were conducted on the ACC and the RT, with two groups (music training and control group) as between-subject variables and two conditions (repeat and switch trials) as within-subject variables. The cost switch was calculated by switch trials RT/ACC minus repeat trials RT/ACC. The independent samples *t*-test was used for the switch cost.

### 2.7 EEG recording and analysis

Brain electrical activities were recorded through an elastic cap (Neuroscan, Charlotte, NC, USA) of 32 scalp sites mounted with tin electrodes, with one reference electrode placed on the frontal central aspects (REF) (reference electrode) and a ground electrode on the medial frontal aspect (GRD). The vertical electrooculogram (EOG) (the measured eye movements) was recorded with an electrode placed on the infraorbital area of the left eye. All inter-electrode impedance was maintained below 5 kW during the conductive gel application and throughout the recording.

MATLAB R2014a (MathWorks, Natick, MA, USA) and the EEGLAB toolbox 14.1.1b were used for the data processing. Grand average ERPs for both the repeat and the switch trials were made, while only trials with the correct responses were included in the final averages. To begin, the data were reduced from 1,000 to 256 Hz. EEG data were filtered with a band-pass finite impulse response (FIR) filter between 1 and 45 Hz. The left and the right mastoids were taken as reference sites. The continuous EEG data were divided into trials according to the different marks of stimuli. A baseline correction (−200 to 0 ms) was applied to each trial, and the quality of EEG data was inspected trial by trial. Trials with abnormal fluctuations (too large or too frequent fluctuations) were eliminated, and trials with bad channels were corrected by averaging the amplitudes of their adjacent electrodes. The independent components analysis (ICA) method was applied to the EEG data to remove interference factors (e.g., eye movements, electrocardio) from the data. In the ICA results, components with EOG artifacts and head movement were removed after visual inspections. Based on the topographical distribution of the grand-averaged ERP activities, the ERPs and their time window: N2, 250–360 ms; P3, 360–500 ms; N450, 500–700 ms.

Three repeated-measure ANOVAs [2 (group: music training and control group) × 2 (condition: repeat and switch trials)] were conducted on the N2 and P3 amplitudes, with the group as a between-subjects factor and the condition as a within-subjects factor.

Data analysis was conducted using SPSS 22.0 (IBM, New York, NY, USA), and the Greenhouse–Geisser method was used to adjust *p*-values for sphericity. Multiple comparison corrections were also conducted with Bonferroni correction.

### 2.8 Pearson's correlation analysis

To explore the relationship between the tone-related response inhibition and cognitive flexibility and its neural correlates, Pearson's analysis was used in the present study.

## 3 Results

### 3.1 Behavioral results

#### 3.1.1 The Raven Advanced Progressive Matrices

There was no significant difference in the mean RAPM scores between the music training group (*M* = 9.34, *SD* = 1.54) and the control group (*M* = 9.42, *SD* = 1.47), *t*_(46)_ = 0.19, *p* = 0.85.

#### 3.1.2 Oddball task

**Figure 3:**
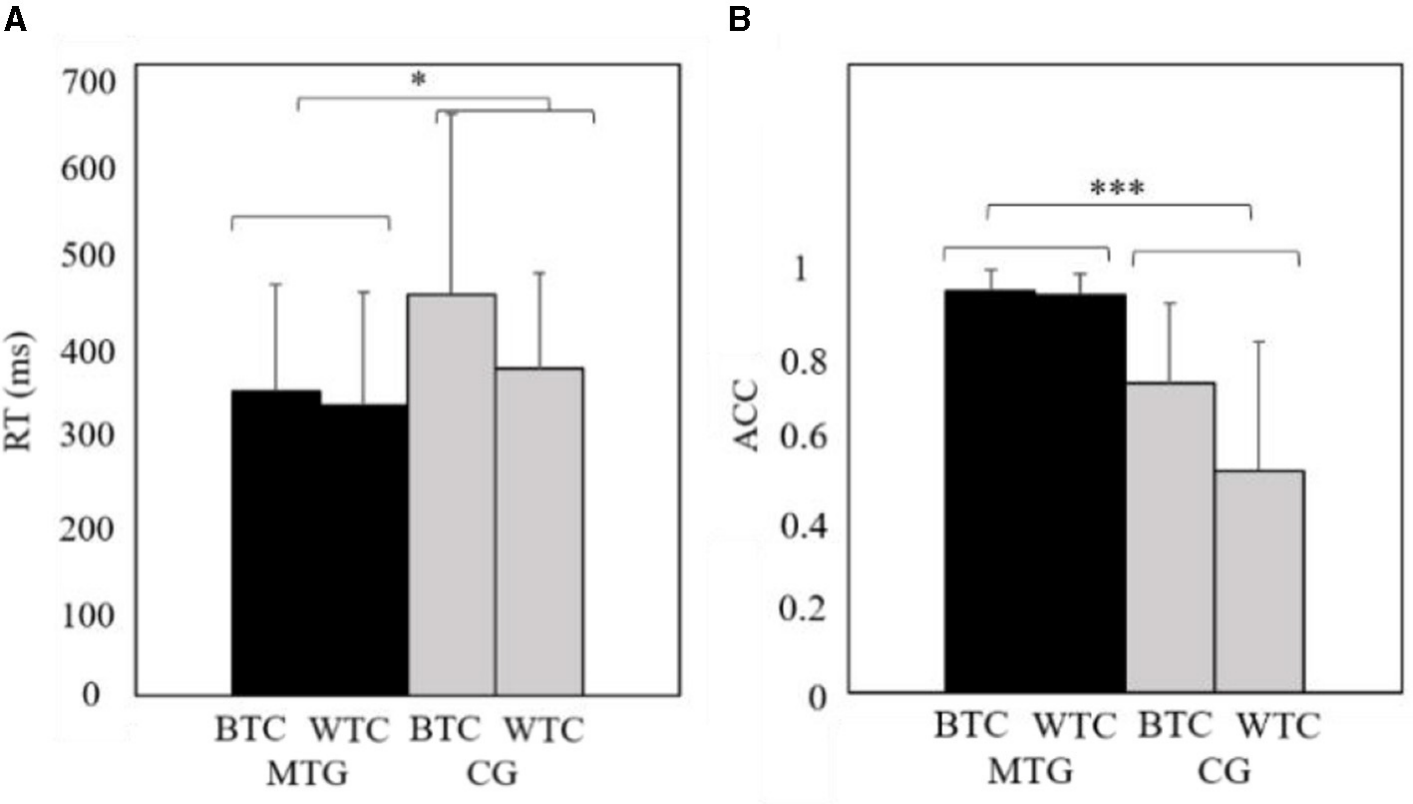
The behavioral results in the oddball task, **(A)** results on reaction time and **(B)** results on accuracy. MTG, the music training group; CG, the control group; BTC, between-tone category; WTC, within-tone category. ^*^p < 0.05, ^***^p < 0.001.

Results on RT (Figure 3A) showed a main effect of group on RT, *F*_(1, 45)_ = 4.62, *p* = 0.04, partial η^2^ = 0.09, such that the RT in the music training group was shorter than that in the control group. Results also showed a main effect of condition on RT, *F*_(1, 45)_ = 4.30, *p* = 0.04, partial η^2^ = 0.09, such that the RT in the between-tone category was greater than that in the within-tone category.

Results on ACC (Figure 3B) showed a main effect of group on ACC, *F*_(1, 46)_ = 57.94, *p* < 0.001, partial η^2^ = 0.56, such that the ACC in the music training group was greater than that in the control group. Results also showed a main effect of condition on ACC, *F*_(1, 45)_ = 8.35, *p* = 0.01, partial η^2^ = 0.16, such that the ACC in the between-tone category was greater than that in the within-tone category. Results also showed a significant interaction between the group and condition, *F*_(1, 45)_ = 7.15, *p* = 0.01, partial η^2^ = 0.14, in that ACC in the music training group was greater than that in the control group in both conditions.

#### 3.1.3 Switch task

**Figure 4:**
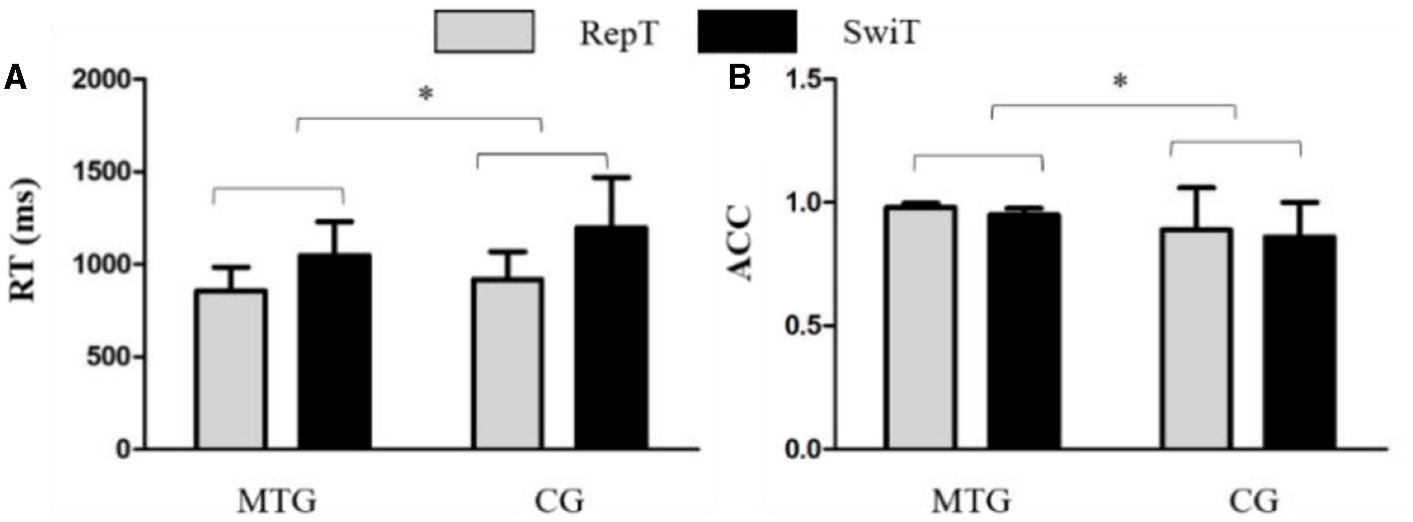
The behavioral results in the switch task, **(A)** results on repeated trials and **(B)** results on switch trials. MTG, the music training group; CG, the control group; RepT, repeat trials; SwiT, switch trials. ^*^p < 0.05.

Results on RT (Figure 4A) showed a main effect of condition on RT, *F*_(1, 46)_ = 141.49, *p* < 0.001, partial η^2^ = 0.76, that the RT in the switch trials was greater than that in the repeat trials. Results showed a main effect of the group on RT, *F*_(1, 46)_ = 4.1, *p* = 0.05, partial η^2^ = 0.08, such that the RT in the control group was greater than that in the music training group. The results also showed an interaction between group and condition, *F*_(1, 46)_ = 5.02, *p* = 0.03, partial η^2^ = 0.10, in which the RT in the control group was greater than that in the music training group in both conditions (music repeat: *M* = 855.13, *SD* = 129.46; music switch: *M* = 1047.29, *SD* = 183.51; control repeat: *M* = 916.82, *SD* = 150.71; control switch: *M* = 1198.15, *SD* = 270.38).

Results on ACC (Figure 4B) showed a main effect of group on ACC, *F*_(1, 46)_ = 8.86, *p* = 0.01, partial η^2^ = 0.16, in which the ACC in the music training group (*M* = 0.97, *SD* = 0.022) was greater than that in the control group (*M* = 0.88, *SD* = 0.020).

Results on RT switch cost showed that the switch cost in the music training group (*M* = 192.15, *SD* = 85.63) was lower than that in the control group (*M* = 281.33, *SD* = 169.06), *t*_(46)_ = 2.24, *p* = 0.03. We did not find the difference between the two groups in the ACC switch cost, *t*_(46)_ = 0.35, *p* = 0.73.

### 3.2 ERP results of the switch task

#### 3.2.1 N2

Results on N2 showed a main effect of condition, *F*_(1, 46)_ = 24.49, *p* = 0.02, partial η^2^ = 0.12, *post-hoc t*-test showed that N2 amplitude in the repeat trials was greater than that in the switch trials. The main effect of the group is marginally significant, *F*_(1, 46)_ = 3.34, *p* = 0.07, partial η^2^ = 0.07, and the N2 amplitude in the music training group was marginally greater than that in the control group.

#### 3.2.2 P3

Results on P3 showed a main effect of group, *F*_(1, 46)_ = 13.68, *p* < 0.001, partial η^2^ = 0.23. The P3 amplitude in the control group was greater than that in the music training group. We did not find any significant difference between the two conditions, *p* = 0.58.

#### 3.2.3 N450

Results on N450 showed a main effect of group, *F*_(1, 46)_ = 14.10, *p* < 0.001, partial η^2^ = 0.24, the N450 amplitude in the music training group was greater than that in the control group. We did not find any significant difference between the two conditions, *p* = 0.55.

### 3.3 Pearson correlation analysis

Pearson correlation analysis showed that the RT in the between-tone category was positively related to the RT in the switch trials (*r* = 0.38, *p* = 0.01) and that in the repeat trials (*r* = 0.36, *p* = 0.01). The RT in the within-tone category was positively related to the RT in the switch trials (*r* = 0.32, *p* = 0.03) and the RT switch cost (*r* = 0.33, *p* = 0.02). In addition, ACC in the between-tone category was negatively related to the N2 in the repeat trials (*r* = −0.48, *p* < 0.001), P3 in the repeat (*r* = −0.55, *p* < 0.001) and switch trials (*r* = −0.34, *p* = 0.02), N450 in the repeat (*r* = −0.54, *p* < 0.001) and switch trials (*r* = −0.28, *p* = 0.05); ACC in the within-tone category was negatively related to the P3 in the repeat (*r* = −0.33, *p* = 0.03) and switch trials (*r* = −0.35, *p* = 0.02), N450 in the repeat (*r* = −0.37, *p* = 0.01) and switch trials (*r* = −0.40, *p* = 0.01).

## 4 Discussion

### 4.1 The oddball task

The outcomes of the oddball task analysis indicated that the response time (RT) of the group with music training was significantly shorter than that of the control group. This observation suggests an enhanced capacity for eliciting quicker responses, implying an enhanced ability to rapidly perceive and react to stimuli. These findings align with prior research conducted by Marie et al. (2011). Moreover, in terms of accuracy (ACC), the participants in the music training group demonstrated superior performance compared to those in the control group, both within the same tone category and across different tone categories. It is worth noting that previous research by Burnham et al. (2015) reported conflicting results. Specifically, Burnham et al. found that individuals with music training displayed a significantly enhanced ability to discriminate within-tone categories compared to the control group. However, the disparity in their ability to distinguish between-tone categories did not reach statistical significance. This discrepancy may be attributed to the distinctiveness of the tones used in the study, where both musically trained and untrained individuals could proficiently identify them during the testing process. It is evident that the capacity to differentiate within-tone categories demands a heightened sensitivity to tonal nuances and a skill that can be refined through music training. This is consistent with the notion that music training contributes to improved precision in minor tonal distinctions and significantly enhances one's tonal judgment abilities, as concluded by the findings of Wu et al. (2015). Consequently, these observations corroborate the disparities in accuracy revealed in our study between the music group and the control group in tasks involving within-tone categories as opposed to those encompassing between-tone categories. In the light of the outcomes derived from the oddball task, we draw the conclusion that individuals with music training exhibit superior performance in this task, as indicated by their reduced response times and elevated accuracy in both experimental conditions compared to individuals without music training. This underscores their heightened proficiency in discerning subtle tonal distinctions at an accelerated pace, as opposed to their non-musically trained counterparts.

### 4.2 The switch task

The music group showed shorter reaction times in both the repeated and the switching tasks, while the advantage in the switching tasks was more pronounced. This could be attributed to the fact that individuals undergoing music training need to coordinate their eyes, hands, and brain during training and recognize notes and other visual information, which requires good cognitive and operational abilities (Norton et al., 2005). As a result of long-term training, they are better equipped to solve and handle task transitions when encountering task conflicts, thus leading to the shorter reaction time observed in the music group than in the control group. For the comparison between switching tasks and repeated tasks, both the music group and the control group have longer RT during switching tasks, indicating that individuals consumed fewer cognitive resources during repeated tasks. Not surprisingly, the ACC of the music group was higher than that of the control group. This could be attributed to the fact that musicians are more likely to learn to pair pitch patterns with word meanings, which is similar to learning vocabulary tones (Wong and Perrachione, 2007). Through music training, they become more sensitive to the vocabulary and intonation of language and can judge tonal changes more accurately. Combining the results of ACC with RT, we found that individuals in the music group have a higher accuracy while making quicker judgments, which verified the advantages of individuals in the music training group over the control group in terms of the switch task.

The difference between RT and ACC between repeated and switching attempts is referred to as the switching cost, and the smaller the switching cost, the stronger the cognitive flexibility. In terms of the cost switch of RT, the switching cost of the music group is lower than that of the control group. The results indicated that people who have undergone music training have higher cognitive flexibility, which remains consistent with the previous research (Biasutti and Mangiacotti, 2018).

Previous studies have mostly shown that individuals who receive music training have unique cognitive advantages in the brain. Executive function can affect a series of brain activities and explain the differences in cognitive function between music-trained and non-music-trained individuals (Hannon and Trainor, 2007). The researchers have also shown that music training can promote the transfer of skills through the regulation of inhibitory control and other functions (Moreno and Farzan, 2015). In our study, in both the oddball task and the switch task, individuals in the music group showed high accuracy and shorter reaction time, which reflected their ability to distinguish and respond at a faster rate and have stronger cognitive flexibility when performing random or switching tasks more flexibly.

### 4.3 Electrophysiological indicators

**Figure 5:**
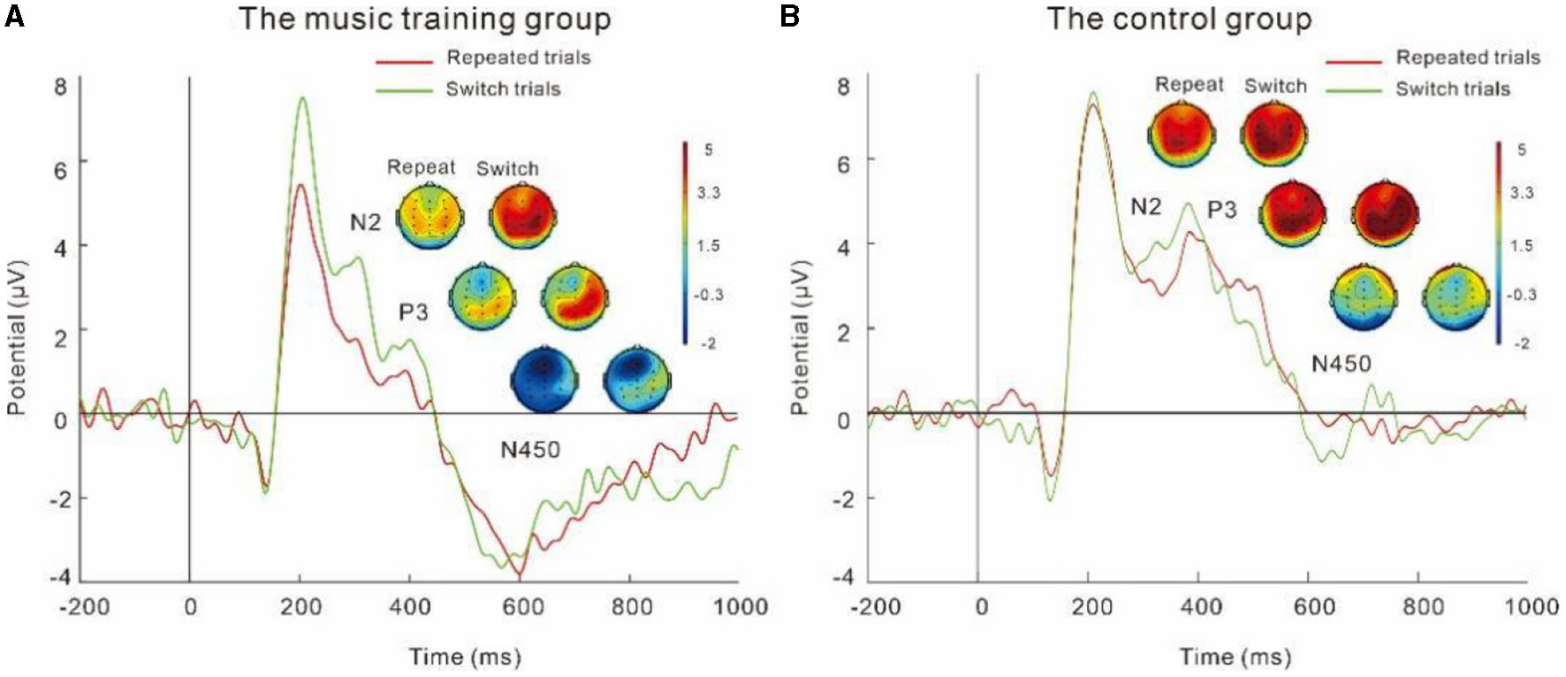
**(A, B)** Grand-average ERPs for repeated and switch trials in the music training and control groups.

The present study found that the music group elicited greater N2 amplitudes during the switch task than the control group (see Figure 5). The main effect of the group is marginally significant, *F*_(1, 46)_ = 3.34, *p* = 0.07, partial η^2^ = 0.07, and the N2 amplitude in the music training group was marginally greater than that in the control group. Individuals in the music group seemed to be able to mobilize higher cognitive monitoring when faced with task switching or repetition, thereby exhibiting higher N2 amplitudes (Getzmann et al., 2015). The current findings also reveal that the amplitude of the N2 component in the repeated trials was greater in magnitude than that in the switch trials. Prior research has predominantly postulated that during task-switching exercises, individuals engage in heightened cognitive monitoring to manage external transitions effectively, resulting in increased N2 amplitudes. This, however, contradicts the outcomes of our own investigation. Our study observed that contrary to the previous study, the amplitude of the N2 component was actually lower in both the music-trained and control groups during the switching trials as opposed to the repeated trials. These findings suggest that the act of switching between tasks decreases the amplitude of the N2 component. It is important to note that, in general, switching trials tend to elevate the likelihood of participants making errors in comparison to the more repetitive trials. Moreno et al. (2014) discovered that good inhibition performance was associated with an increase in N2 wave amplitude, implying that an increase in accuracy is related to an increase in N2 wave amplitude. In the present study, both groups exhibited lower N2 amplitudes during switching trials. This could be owing to the participants' decreased cognitive monitoring capacity during tasks with greater difficulty, making it difficult to concentrate or easily mobilize cognitive flexibility functions, resulting in a reduced amplitude of N2 compared to the repeated trials.

P3 represents various cognitive abilities (Chu et al., 2015), including discrimination, exercise preparation, reaction inhibition, and behavioral monitoring. Previous studies have demonstrated that individuals with music training typically have higher P3 amplitudes, which reflect their better cognitive abilities (Collins and Koechlin, 2012). However, our results showed that the P3 amplitude of the control group was greater than that of the music group, which has also been confirmed by Hao et al. (2023). During their selecting tasks, individuals in the control group put in more cognitive effort, demonstrating that the control group needed more monitoring and inhibitory abilities in order to respond correctly to the switch task. In contrast, the P3 amplitude of the music group is slightly smaller. The ability of the music group to distinguish tones is trained through music training, so when performing switch task, it is unnecessary for them to employ more cognitive monitoring ability to make accurate and fast judgments on the task, which showed that individuals in the music group demonstrated greater cognitive flexibility and can handle external repetitive or switching stimuli more easily. The effortless switching can be attributed to their proficiency in improvisation and their capacity to adapt to various musical contexts. Improvisation demands the ability to generate novel ideas, transition between various musical patterns, and adjust to changing musical cues. These factors of task switching are integral to cognitive flexibility (Donnay et al., 2014).

N450 is usually detected in conflicting reactions. Research has shown that N450 is mainly related to stimulus conflicts at the level of stimulus processing (Szucs and Soltész, 2012). In the present study, we observed that the N450 amplitudes in the music-trained group were greater than those in the control group. This difference may be attributed to the fact that individuals who have undergone extensive music training are adept at swiftly and effectively deploying conflict monitoring capabilities when confronted with tonal discrepancies. This heightened capacity is thought to stem from their extensive experience in handling musical conflicts. As a result, they are able to transfer these conflict resolution skills, acquired through musical training, to effectively address linguistic and tonal conflicts (Strait and Kraus, 2011). In contrast, individuals who lack music training do not possess the same reservoir of experience and the ability to transfer these skills. Consequently, when they encounter conflicting information, their comparatively diminished cognitive monitoring and conflict suppression abilities are manifested through smaller N450 amplitudes.

Our results revealed that the RT between tones was related to the RT of switch and repeat trials. When faced with selection difficulties, certain individuals may have specific patterns of fast or slow selection, resulting in similar performance when faced with reaction tasks. There was, however, a positive relationship between RT in the within-tone category and the RT in the switch trials and the switch cost of RT. This suggests that when confronted with challenging tasks, individuals who can swiftly notice and respond to them have strong cognitive monitoring ability and cognitive flexibility, reflected by the switch cost. Furthermore, when conflicting stimuli arise, N2 is in charge of monitoring them, P3 is in charge of allocating and processing cognitive resources, and N450 is in charge of adapting and resolving conflicting stimuli (Liu et al., 2019). Our findings revealed that increases in N2, P3, and N450 were adversely linked with ACC in repeated tasks, indicating a reduction in control over a series of conflicts when a stimulus arises. Perhaps it is because individuals do not need to exert a considerable level of cognitive control to obtain ACC improvement in simple between-tone categorization trials. We speculate that this is due to the fact that when an individual ACC declines during switching trials; they desire to increase ACC by increasing cognitive engagement, which results in an increase in the amplitudes of P3 and N450. This would also explain the negative association between the ACC for the within-tone category and P3 and N450 amplitudes.

There are certain limitations to our research. To begin, our experiment was a cross-sectional study with no individual longitudinal surveillance. Second, we lack a large enough sample size to differentiate the types of music training individuals received, such as musical instruments or vocal training. Third, the study does not account for individual differences, including childhood environment or economic status. To overcome these constraints, future researchers can combine horizontal and longitudinal studies, incorporate more independent factors and control them. Furthermore, the music training group can be subdivided further to investigate the impact of different types of music training on individual cognitive flexibility.

## 5 Conclusion

Music training has been demonstrated in studies to be advantageous in the development of an individual's cognitive flexibility. The group with music training exhibited higher N2 and N450 amplitudes, along with lower P3 amplitudes during switch trials. These findings suggest that individuals who have received music training possess an advantage in perceiving musical tones and can engage their cognitive recognition abilities in a more adaptable manner. When exposed to specific stimuli, they require less cognitive effort. In essence, music education can enhance executive function, such as cognitive flexibility, in individuals. Those with improved executive function tend to perform better across a range of tasks. Therefore, early music education for children holds significant importance, and society should devote sustained attention to fostering children's musical education.

## Data availability statement

The raw data supporting the conclusions of this article will be made available by the authors, without undue reservation.

## Ethics statement

The studies involving humans were approved by the Ethics Committee of Southwest University. The studies were conducted in accordance with the local legislation and institutional requirements. The participants provided their written informed consent to participate in this study.

## Author contributions

JH: Conceptualization, Data curation, Investigation, Methodology, Resources, Writing – review & editing. YZ: Investigation, Methodology, Writing – review & editing. YP: Writing – original draft, Writing – review & editing. YJ: Writing – original draft, Writing – review & editing. YL: Conceptualization, Data curation, Formal analysis, Methodology, Resources, Writing – original draft, Writing – review & editing. HL: Data curation, Resources, Writing – review & editing. JL: Investigation, Resources, Writing – review & editing. MZ: Conceptualization, Funding acquisition, Project administration, Supervision, Writing – review & editing.
